# Chromatin state dynamics during the *Plasmodium falciparum* intraerythrocytic development cycle

**DOI:** 10.1186/s12864-025-12455-3

**Published:** 2026-01-07

**Authors:** Alan S. Brown, Manuel Llinás, Shaun Mahony

**Affiliations:** 1 Center for Eukaryotic Gene Regulation, Department of Biochemistry & Molecular Biology, The Pennsylvania State University, University Park, PA 16802 USA; 2 Huck Center for Malaria Research, The Pennsylvania State University, University Park, PA 16802 USA; 3 Department of Chemistry, The Pennsylvania State University, University Park, PA 16802 USA

**Keywords:** Plasmodium falciparum, Gene regulation, Chromatin, Chromatin state, Histone modifications, Transcription factors

## Abstract

The interdependence of chromatin states and transcription factor (TF) binding in eukaryotic genomes is critical for the proper regulation of gene expression. In this study, we explore the connection between TFs and chromatin states in the human malaria parasite, *Plasmodium falciparum*, throughout its 48-hour asexual intraerythrocytic developmental cycle (IDC). Most *P. falciparum* genes are expressed in a periodic manner during the IDC, accompanied by dynamic shifts in histone modifications and chromatin accessibility. Leveraging genome-wide profiles of chromatin accessibility, histone modifications, and Heterochromatin Protein 1 (HP1) occupancy, we characterize chromatin state dynamics during the IDC. Our results indicate that several chromatin states remain stable throughout the lifecycle, while others are dynamic and are linked to gene activation or repression. We further characterize chromatin state dynamics at the genome-wide DNA binding sites for a selection of *Plasmodium* TFs, allowing us to group TFs according to their chromatin preferences. By correlating changes in chromatin accessibility, histone modifications, and TF binding, we provide a global overview of the chromatin state dynamics that coordinate *P. falciparum* asexual blood stage development.

**Supplementary Information:**

The online version contains supplementary material available at 10.1186/s12864-025-12455-3.

## Introduction

Methods for identifying combinatorial patterns of histone modifications, or “chromatin states”, have been applied to characterize regulatory elements across several multicellular eukaryotic species, including *Arabidopsis thaliana*, *Caenorhabditis elegans*, *Drosophila melanogaster*, mouse, and human [1–8]. By integrating multiple datasets into categorical variables representing co-occurring patterns of histone modifications, chromatin states distill complex epigenetic information into more easily interpretable regulatory categories. These categories reflect the underlying biology driving gene expression. Chromatin state analysis has been effectively used in large scale genomic research projects to annotate key regulatory features across many cell types and conditions [7, 8]. For example, the Encyclopedia of DNA Elements (ENCODE) and other mammalian epigenomic consortium studies have used ChromHMM to define chromatin states by applying a hidden Markov model to binarized chromatin signals (i.e., presence/absence calls), thereby annotating potential regulatory regions [1, 2, 7–11]. Similarly, Segway, a dynamic Bayesian network-based approach for segmenting genomic data, was also deployed by ENCODE [9, 11–13]. The integrative and discriminative epigenome annotation system (IDEAS), which uses a 2D segmentation approach to define consistent chromatin states across genomic locations and cell types, has been used to integrate epigenomics datasets across human and mouse hematopoiesis [14–18].

Chromatin state analyses have not been widely used to characterize regulatory features in single-celled eukaryotes such as the human malaria parasite, *Plasmodium falciparum*. The lifecycle of *P. falciparum* includes a 48-hour asexual intraerythrocytic developmental cycle (IDC), during which the parasite population expands exponentially in red blood cells leading to the clinical symptoms of malaria. During the IDC, the parasite transitions through three distinct morphological stages inside a red blood cell – rings, trophozoites, and schizonts – before egressing as merozoites to invade new red blood cells [19]. Gene mRNA abundance is highly dynamic during the IDC, with most genes displaying a periodic transcript abundance pattern that correlates with specific stages of development [20–23].

While several studies have characterized individual histone modifications, histone variants, chromatin accessibility, transcription factor binding, or transcript abundance during the IDC [24–34], our understanding of how combinations of these chromatin features relate to regulatory dynamics remains incomplete. A single previous study has profiled chromatin states based on three histone modifications in parasites that were transitioning through the merozoite stage [35], but this did not fully describe chromatin state dynamics throughout the IDC. We also previously demonstrated that *P. falciparum* TF binding site selection is impacted by chromatin features [36]. Thus, a more complete characterization of chromatin state dynamics is crucial for understanding the tightly regulated asexual developmental cycle of *Plasmodium* parasites.

The *P. falciparum* genome has several unique features that make it uncertain whether chromatin state principles derived from other species are directly transferable. Its genome is extremely A/T-rich, with an average A/T content of 80.6% and up to 90% in non-coding regions [37–40]. Although *Plasmodium* parasites encode all of the canonical histones [41, 42] except the linker histone H1 [37, 43], the *Plasmodium* histone H2A sequences are somewhat divergent from other eukaryotes [44]. Additionally, *Plasmodium* encodes the histone variant H2A.Z, which is commonly found at promoters and enhancers in mammalian species [45] and is associated with actively transcribed genes in accessible chromatin in mammals and *Plasmodium* parasites [24, 33, 46].

Other properties of *Plasmodium* nucleosomes also differ from those of humans and yeast, including reduced stability under varying conditions, and decreased effectiveness at binding GC-rich DNA [39]. Importantly, some histone modifications have atypical associations with regulatory activities in *P. falciparum* compared to other eukaryotes. For instance, H3K36me3, which typically marks active transcription in *Drosophila*, mouse, and humans [47, 48], is instead associated with gene silencing in *P. falciparum* [28, 49]. H3K4me1 has been associated with both poised chromatin states and active regulatory elements in *Plasmodium* [29, 50], whereas in animal genomes it primarily marks distal enhancer elements [51, 52]. These differences in chromatin associations highlight the need for a *Plasmodium*-specific understanding of chromatin state dynamics.

Studies of individual epigenomic features point to a highly dynamic chromatin landscape across different stages of the *P. falciparum* IDC. For example, chromatin accessibility increases significantly after the ring stage and peaks during the trophozoite or early schizont stages, correlating with increased mRNA abundance [30, 34, 53]. H3K4 methylation increases after the ring stage, while H3K27 acetylation is highest during the ring stage, and H3K9 acetylation predominates in the trophozoite stage [24, 27, 54]. The highly dynamic expression program is tightly controlled by a relatively small number of transcription factors (TFs); there are only 73 predicted TFs encoded in the *Plasmodium* genome [55–57], including 30 members of the apicomplexan APETALA2 (ApiAP2) family that have no known homologs in humans [58]. Understanding how this limited TF repertoire interacts with the dynamic chromatin landscape is essential for deciphering the regulatory mechanisms that drive developmental transitions in *Plasmodium* parasites.

In this study, we comprehensively characterize chromatin state dynamics throughout the *P. falciparum* IDC by integrating data on chromatin accessibility, histone modifications, and Heterochromatin Protein 1 occupancy. We further analyze these chromatin states at the binding sites of key ApiAP2 transcription factors, allowing us to group TFs according to chromatin state preferences. By correlating changes in chromatin accessibility, histone modifications, and TF binding, we provide insights into the gene regulatory network that coordinates asexual blood stage development of the malaria parasite during infection of the human host.

## Results

### Defining chromatin states during the *P. falciparum* intraerythrocytic development cycle

To define chromatin regulatory landscapes during the *P. falciparum* IDC, we compiled epigenomic data from multiple published sources (see Methods). Since these experiments were generated by individual labs rather than a coordinated effort, only a subset of signals has been profiled across consistent developmental timepoints. We therefore focused on the three main stages of the IDC: ring stage (10 h post invasion (hpi)); trophozoite stage (30 hpi); and schizont stage (40 hpi). Our analysis included seven histone modifications/variants with data available for all three stages: H3K4me1, H3K4me3, H3K9me3, H3K9ac, H3K18ac, H3K27ac, and H2A.Z, along with Assay for Transposase-Accessible Chromatin using sequencing (ATAC-seq) data measuring chromatin accessibility. We also incorporated Chromatin Immunoprecipitation followed by sequencing (ChIP-seq) data for Heterochromatin Protein 1 (HP1), a known indicator of heterochromatic regions.

**Fig. 1:**
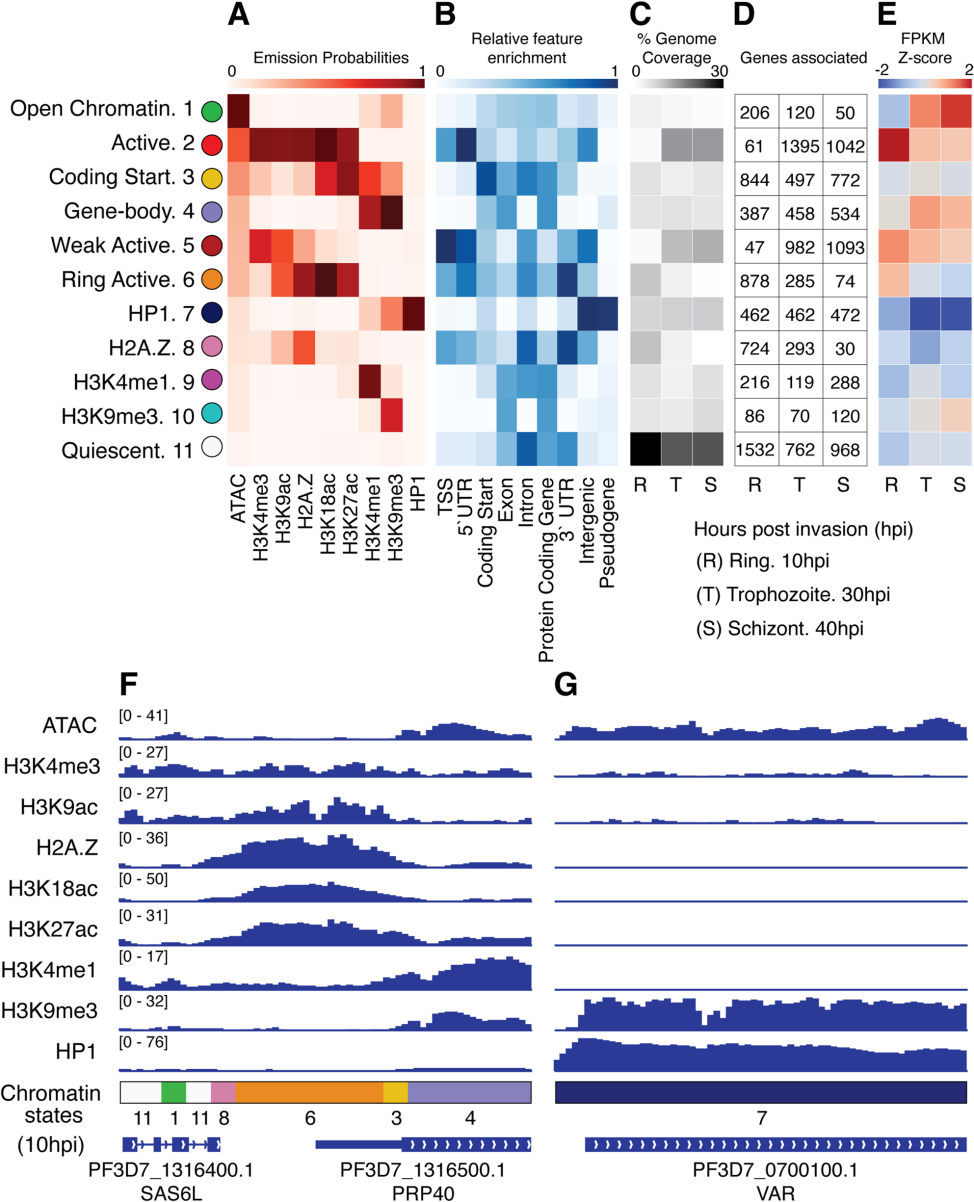
Chromatin states in the *P. falciparum* IDC and associated features. **A** Hidden Markov Model emission probabilities (i.e., the probability of seeing the mark given the chromatin state) for each of the 11 chromatin states discovered by ChromHMM. **B** Relative enrichment of each state at a selection of genomic features. **C** The genomic coverage of each state at each timepoint: R = ring stage (10hpi), T = trophozoites (30hpi), and S = schizont stage (40hpi). **D** The number of genes for which each state is located at the start of the coding sequence at each timepoint. **E** Z-score values for the average mRNA abundance per chromatin state, measured via RNA-seq Fragments Per Kilobase of transcript per Million mapped reads (FPKM) values. **F** 10hpi chromatin signal tracks and associated chromatin states in a euchromatic locus at coordinates Pf3D7_13_v3:683,666–686,994. Numbers in square brackets on each chromatin track denote the y-axis scales in counts per million mapped reads (CPM) for each signal. **G** 10hpi chromatin signal tracks and associated chromatin states in a heterochromatic locus at coordinates Pf3D7_07_v3:20,068 − 23,396. See Supplemental Figure S1 for additional chromatin track examples

We used ChromHMM [2] to identify chromatin states across the *P. falciparum* genome at a resolution of 200 bp for our chromatin state bins. We determined 11 states to be optimal after testing various state numbers (see Methods). Our results identified the probabilities of observing each epigenetic signal in genomic regions covered by each of the 11 states (Fig. 1A). We also determined the relative chromatin state enrichment at various structural genome features (Fig. 1B), their genome-wide prevalence (Fig. 1C), and their occurrence at genes (Fig. 1D) across all three developmental timepoints. For clarity and ease of discussion, we refer to the chromatin states by descriptive names based on prominent associations or histone mark features, alongside their numerical labels.

Several identified chromatin states parallel those observed in other eukaryotic systems, including those characterized in the human genome by the ENCODE and NIH Roadmap Epigenomics Mapping Consortium [9–11]. “Active” state 2, which is characterized by a combination of chromatin accessibility, H3K4me3, H2A.Z, and various histone acetylation marks, is highly enriched at transcription start sites (TSSs), similar to active promoter states in vertebrates and *Drosophila* [1, 7, 59, 60]. Other states that include H3K4me3 and/or H2A.Z marks (“Weak Active” state 5, “Ring Active” state 6, and “H2A.Z” state 8) also show associations with TSSs and 5′ untranslated regions (5′ UTRs). “Coding Start” state 3, which is enriched for H3K4me1, H3K27ac, and ATAC-seq signals, resembles enhancer-associated signatures in vertebrates [1, 60]. However, unlike vertebrate genomes, where these marks identify distal enhancers, the *P. falciparum* “Coding Start” state 3 is more highly associated with coding start regions and gene bodies than with intergenic regions. “Coding Start” state 3 may thus be analogous to an H3K4me1-enriched chromatin state that is observed flanking TSSs in vertebrate genomes [1, 60]. “Quiescent” state 11 features an absence of measured chromatin signals and is consistent with the ENCODE designated chromatin state that covers up to 80% of the human genome [1, 61]. This state is also the most frequently occurring in *P. falciparum*, appearing in 25–30% of the measured genomic windows (Fig. 1C). “Open Chromatin” state 1, displaying only ATAC-seq enrichment, is seen relatively rarely in *P. falciparum*, although it is analogous to a similar chromatin state observed in the human genome [8].

While several *P. falciparum* chromatin states have analogs in vertebrate systems, the H3K9me3-associated states show some unique properties. In the human genome, H3K9me3 typically marks repressed heterochromatin. Concordantly, “HP1” state 7 in our analysis is characterized by enrichment of both H3K9me3 and HP1 and likely represents heterochromatic regions. However, “Gene-body” state 4 displays enrichment of both H3K9me3 and H3K4me1, without HP1, and is associated with exonic sequences. Similarly, states displaying only H3K4me1 (“H3K4me1” state 9) or only H3K9me3 (“H3K9me3” state 10) are also associated with exons. Thus, *P. falciparum* may use H3K9me3 and H3K4me1 to mark genomic processes in a manner that differs from vertebrate genomes.

Examining the relationships between chromatin states and mRNA abundance, we find that three of the four TSS-associated states (“Active” state 2, “Weak Active” state 5, and “Ring Active” state 6) correlate with higher mRNA abundance levels (Fig. 1E), consistent with “active” TSS states in human. The exception is the H2A.Z-enriched “H2A.Z” state 8, which is associated with lower mRNA levels and is differentiated from other TSS-associated states by a relative lack of histone acetylation signals. Interestingly, the prevalence of TSS-associated states varies dramatically across the IDC. Chromatin states “Ring Active” state 6 and “H2A.Z” state 8 are common during the ring stage (11.6% and 12.1% of genomic regions, respectively) but become rare at later timepoints. Conversely, chromatin states “Active” state 2 and “Weak Active” state 5 are rare during the ring stage, but each mark the start of over 1,000 genes during trophozoite and schizont stages.

As expected, the putative heterochromatin state (“HP1” state 7) correlates with decreased mRNA abundance (Fig. 1E) and is stably associated with approximately 10% of the genome throughout the IDC. This state is primarily found in telomeric, subtelomeric, and centromeric regions, and encompasses most of the clonally variant *var* and *rifin* genes involved in immune system evasion through antigenic variation [62]. Most of these genes are constitutively repressed, with only a subset (variable across a population of cells) gaining high mRNA abundance levels [63]. Finally, “Open Chromatin” state 1 (ATAC-seq only) is associated with low levels of mRNA in the ring stage, but higher levels in trophozoite and schizont stages, potentially indicating a “poised” regulatory signature, though its prevalence decreases during the life cycle (from 4.8% genome coverage in ring stage to only 1.8% in schizonts). Together, the 11 chromatin states provide a comprehensive annotation of the *P. falciparum* chromatin landscape – from active promoters to repressive heterochromatin including some unique combinations of epigenetic marks (e.g., the H3K4me1/H3K9me3 “Gene Body” state 4). Chromatin tracks allow direct visualization of the underlying features resulting in associated chromatin state calls (Fig. 1F and G, Supplemental Fig. S1).

### Chromatin states are temporally dynamic during the *P. falciparum* IDC

**Fig. 2:**
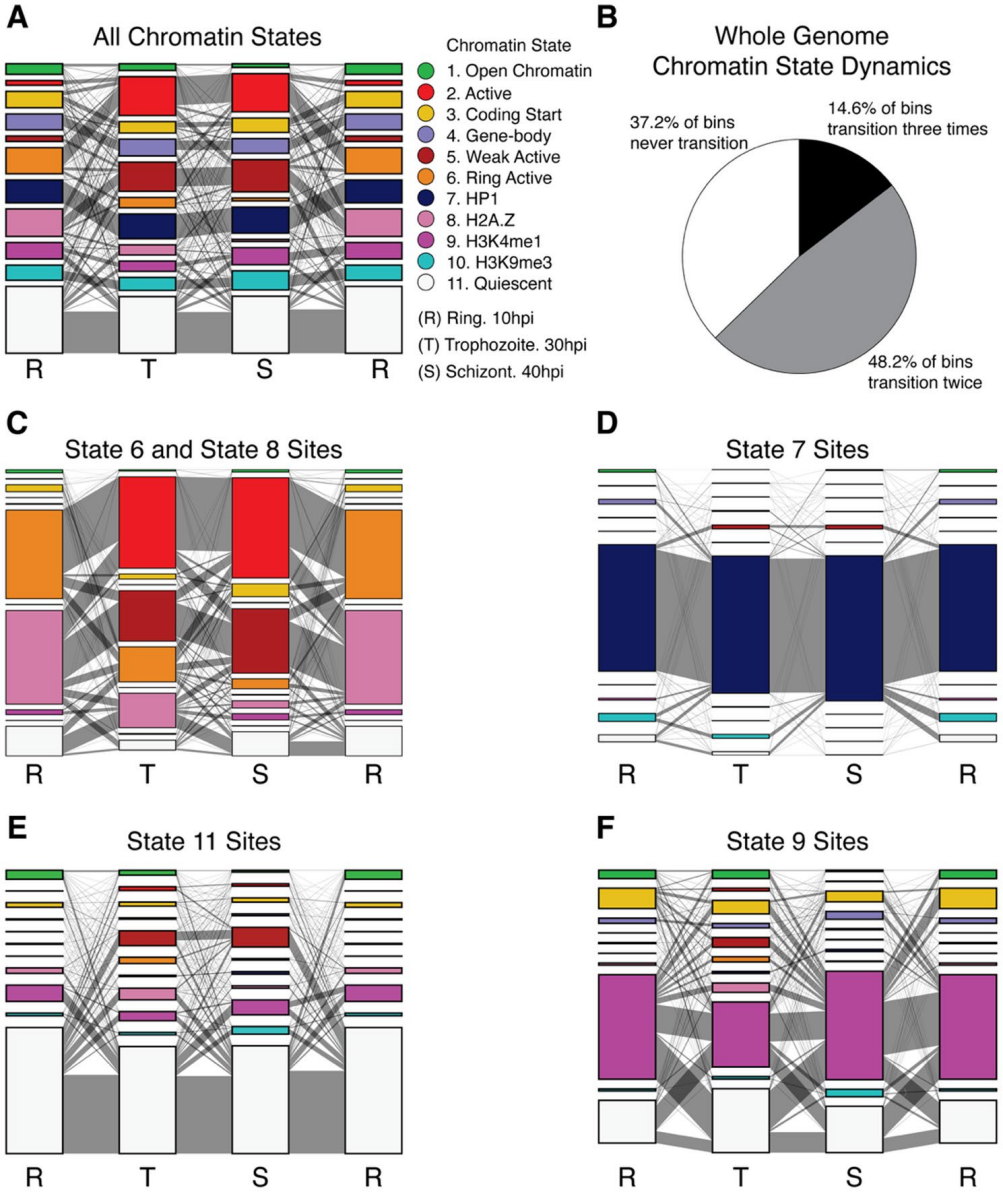
Chromatin state dynamics during the *P. falciparum* IDC. **A** Chromatin state transitions across all 200 bp genomic bins. Stacked bar graphs show the relative proportions of the 11 chromatin states during 10hpi ring (R), 30hpi trophozoite (T), and 40hpi schizont (S) stages. The width of the connecting gray lines between stages represents the number of bins transitioning between each pair of chromatin states. Ring stage is displayed twice to illustrate the full developmental cycle from schizont back to ring stage. The visualization format is maintained in panels C)-F), with each panel focusing on a specific subset of genomic bins to illustrate different aspects of chromatin state dynamics. **B** Proportion of genomic bins that change states across the IDC. “Never transition” encompasses bins that maintained the same state across all 3 timepoints. “Transition twice” represents bins that change to a different state and then revert to their original state. “Transition three times” indicates bins that display a different state at each of the three timepoints. **C** Chromatin state transitions for bins displaying the “Ring Active” state 6 or “H2A.Z” state 8 at any timepoint. **D** Chromatin state transitions for bins displaying the heterochromatin-associated “HP1” state 7. **E** Chromatin state transitions for bins that are labeled “Quiescent” state 11 in at least one timepoint. **F** Chromatin state transitions for bins displaying the “H3K4me1” state 9

Previous work in human and model organisms has demonstrated that chromatin states can change rapidly over developmental time, reflecting underlying dynamics in gene expression and regulatory mechanisms [64–66]. Building on our stage-specific chromatin state analyses, we evaluated the degree to which chromatin states change at individual genomic bins (200 bp) throughout the *P. falciparum* IDC (Fig. 2A). Approximately two-thirds of all genomic bins display at least one change in chromatin state across the three developmental stages examined (Fig. 2B), indicating highly dynamic chromatin behavior during the *P. falciparum* IDC.

As previously noted, the various TSS-associated chromatin states (“Active” state 2, “Weak Active” state 5, “Ring Active” state 6, and “H2A.Z” state 8) each undergo shifts in global abundance during the IDC. Investigating transitions to or from “Ring Active” state 6 and “H2A.Z” state 8, we observed a clear shift in chromatin profiles between the ring stage and the two later trophozoite and schizont stages which are more transcriptionally active (Fig. 2C). Specifically, “Ring Active” state 6 and “H2A.Z” state 8 have a clear progression into “Active” state 2 and “Weak Active” state 5 at later timepoints. Of the genomic bins displaying “Ring Active” state 6 during the ring stage, 71.9% transition to state 2 in the trophozoite stage. Similarly, bins with the H2A.Z-enriched “H2A.Z” state 8 at ring stage transition to “Weak Active” state 5 (41.9%) or “Active” state 2 (29.2%) in trophozoites. As previously mentioned, “H2A.Z” state 8 is not associated with actively transcribed genes, possibly reflecting the overall lower transcriptional activity in the ring stage [20, 67].

The rapid decrease in global prevalence of states “Ring Active” state 6 and “H2A.Z” state 8 and their transitions can be explained by a global increase in the histone mark H3K4me3. The main differences between states “Active” state 2 and “Ring Active” state 6 and between states “Weak Active” state 5 and “H2A.Z” state 8 are the relative levels of H3K4me3 in their respective emission probabilities (Fig. 1A). Our analyses use ChIP-seq data showing globally low levels of H3K4me3 in ring stage parasites but higher levels in trophozoites and schizonts [24]. Other studies corroborate the low level of H3K4me3 in ring stage parasites [27, 35, 54] and one specifically associates H3K4me3 with active genes only in the schizont stage while pointing to its lack of correlation to mRNA abundance in ring stages [31]. Thus, part of the dynamic behavior we observe in chromatin states may reflect a shift in the types of histone modifications present on the *P. falciparum* genome and their stage-specific roles during the IDC.

The persistence of chromatin states over time varies considerably between states. The most stable state is the heterochromatin-associated “HP1” state 7 (Fig. 2D). Of the genomic bins associated with “HP1” state 7 at any timepoint, 79% maintain “HP1” state 7 across all three timepoints. These stable “HP1” state 7 loci exist primarily in the sub-telomeric and centromeric regions, which remain consistently heterochromatic throughout the IDC. As expected, most genes in these regions are repressed, although a small number of clonally variant genes are expressed non-uniformly at high levels across the parasite population [63].

The most highly represented and second-most stable state is the “Quiescent” state 11. Over 38% of “Quiescent” state 11 loci remain in this state across all three timepoints. On average, 70.5% of the “Quiescent” state 11 sites in a given timepoint remain in “Quiescent” state 11 at the next time point (62.4% from 10hpi to 30hpi, 72.5% from 30hpi to 40hpi, and 76.6% from 40hpi back to 10hpi) (Fig. 2E). Of those loci that transition from the quiescent state to some other state, the most common target state is “H3K4me1” state 9 when transitioning from trophozoite to schizont and schizont to ring. During the ring stage, the most common transition target from the “Quiescent” state 11 is “Weak Active” state 5, followed by “H2A.Z” state 8, and “H3K4me1” state 9. This pattern aligns with the broader trend of ring-stage chromatin states shifting to the transcription-associated “Weak Active” state 5 and the overall dramatic increase in transcriptional activity as the parasite enters the trophozoite stage.

In contrast to the stable heterochromatin state, and aside from the TSS-associated states that display large global prevalence changes, the most dynamic state is the H3K4me1-associated state 9. Of the over 13,000 bins that are annotated as “H3K4me1” state 9 in at least one time point, only about 10% remain in “H3K4me1” state 9 at all measured timepoints. Among bins that change across time, an average of 32.5% of “H3K4me1” state 9 bins transition to “Quiescent” state 11 in the subsequent point (43.6% from 10hpi to 30hpi, 32.2% from 30hpi to 40hpi, and 21.8% from 40hpi back to 10hpi) (Fig. 2F).

Overall, our findings demonstrate that temporal chromatin state dynamics vary dramatically across the IDC. Some states are highly stable, while others undergo large-scale shifts that are associated with changes in mRNA abundance. This range of transcriptional plasticity is particularly evident during the transition from the ring to the trophozoite stage, which reflects the dramatic increase in mRNA abundance and global transcription that is known to accompany this developmental transition [68, 69].

### Chromatin state dynamics are associated with mRNA abundance dynamics

**Fig. 3:**
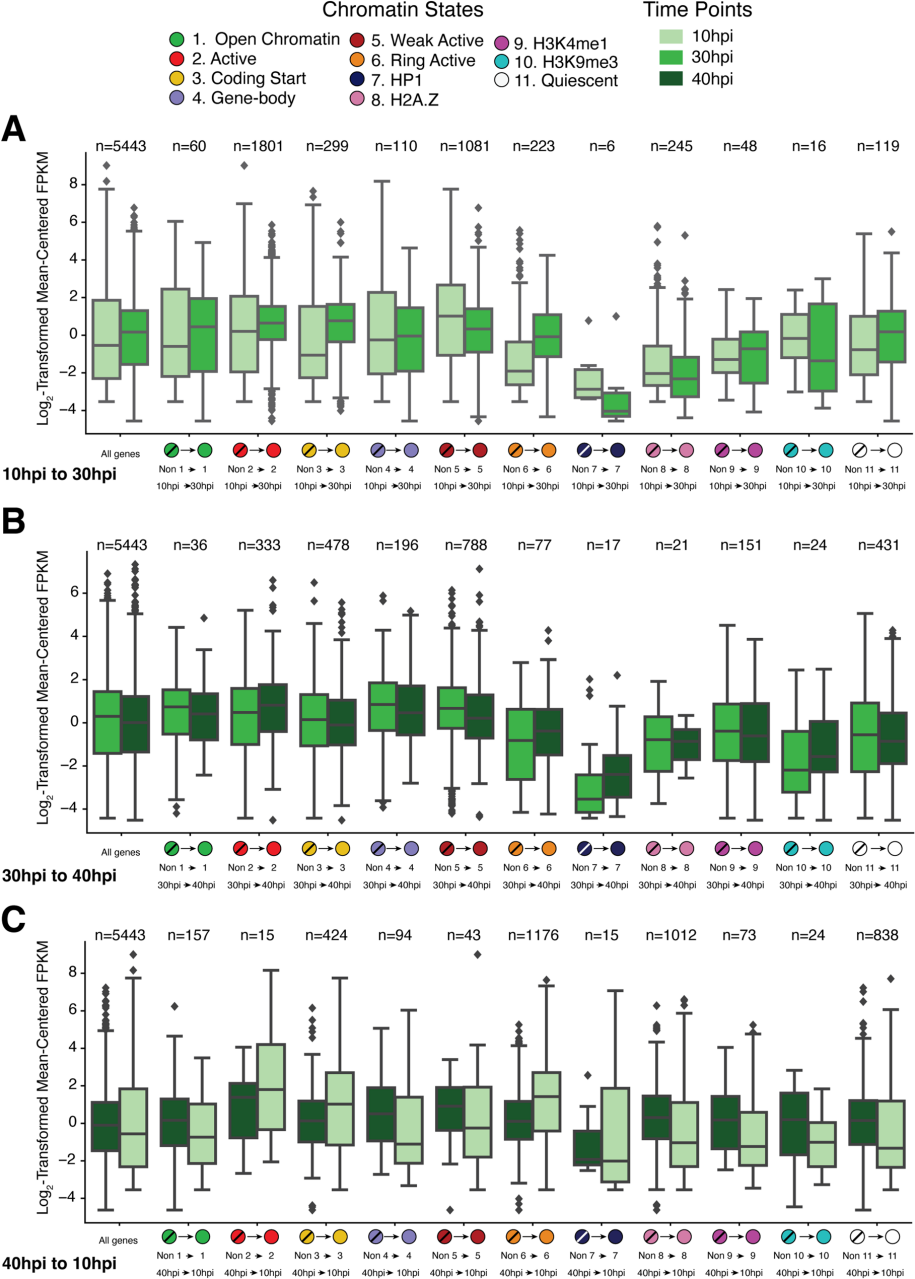
Associations between chromatin state transitions and mRNA abundance dynamics at genes that change states between consecutive pairs of timepoints. Chromatin states are numbered as in Fig. 1. The boxplots display distributions of log_2_-transformed mean-centered FPKM normalized RNA-seq values for groups of genes that display indicated transitions from other states into the labeled state at the labeled timepoints. **A** Chromatin state transitions and normalized RNA-seq values from 10hpi to 30hpi. **B** Chromatin state transitions and normalized RNA-seq values from 30hpi to 40hpi. **C** Chromatin state transitions and normalized RNA-seq values from 40hpi back to 10hpi. See Supplemental Figure S2 for a similar plot showing groups of genes that display no change in chromatin states between consecutive timepoints

While we find that specific chromatin states correlate with either elevated or reduced mRNA abundance (measured by RNA-seq) at individual timepoints (Fig. 1E), both chromatin states and transcriptional activity exhibit highly dynamic and periodic patterns during parasite development. We therefore investigated how temporal changes in chromatin states relate to general shifts in transcript abundance patterns across the parasite asexual cycle. To analyze this relationship, we assigned a single, dominant chromatin state to each gene. Based on evidence that histone modifications near the start codon are most predictive of mRNA abundance levels [70], we assigned each gene the most prevalent chromatin state within a 600 bp window surrounding its start codon (see Methods). We next measured mRNA abundance distributions for genes that change chromatin states between consecutive timepoints and categorized them according to the chromatin state gained (Fig. 3). Interestingly, a subset of genes do not change chromatin states between consecutive pairs of timepoints (Supplemental Fig. S2), and some display no change in chromatin states throughout the IDC (Supplemental Fig. S3). Genes that do not change chromatin states between consecutive timepoints show less change in mRNA abundance than do genes with changing chromatin states.

Genes transitioning into the histone acetylation-rich “Active” state 2 or “Ring Active” state 6 display increases in average mRNA abundance across all three timepoint transitions (Fig. 3). This increase in gene transcript counts is striking for the large group of 1,176 genes transitioning into “Ring Active” state 6 at the ring stage (i.e., from 40hpi to 10hpi). Transitions into “Coding Start” state 3, another acetylation-rich state, also correlated with gains in mRNA abundance. Surprisingly, while “Weak Active” state 5 is associated with higher mRNA abundance levels (Fig. 1E), transitions into this state were associated with reductions in gene transcript counts across all three timepoint transitions (Fig. 3). This suggests that gaining “Weak Active” state 5 might represent a reduction in mRNA abundance levels at genes that otherwise would have high mRNA abundance.

Transitions to the H3K9me3-associated states (i.e., “Gene-body” state 4, “HP1” state 7, or “H3K9me3” state 10) correlated with reductions in mRNA abundance during the 10hpi to 30hpi and 40hpi to 10hpi transitions (Fig. 3). However, this trend was muted, or even reversed, during the transition to the schizont stage (i.e., 30hpi to 40hpi). It should also be noted that relatively few genes gain “HP1” state 7 or “H3K9me3” state 10 during any transition. Transitions into “Open Chromatin” state 1, “H3K4me1” state 9, or “Quiescent” state 11 display mixed, stage-specific associations with transcript abundance changes. On average, a gain of one of these three states is associated with mRNA increase in trophozoites (i.e., 10hpi to 30hpi), a maintenance of transcript levels in schizonts (i.e., 30hpi to 40hpi), and decreased abundance in rings (i.e., 40hpi to 10hpi) – mirroring the global stage-dependent trends in mRNA abundance at those stage transitions. While these associations might point to context-dependent regulatory interactions between chromatin signals and transcript abundance, they may also reflect background associations as average transcript levels across all genes display similar stage-specific dynamics.

**Fig. 4:**
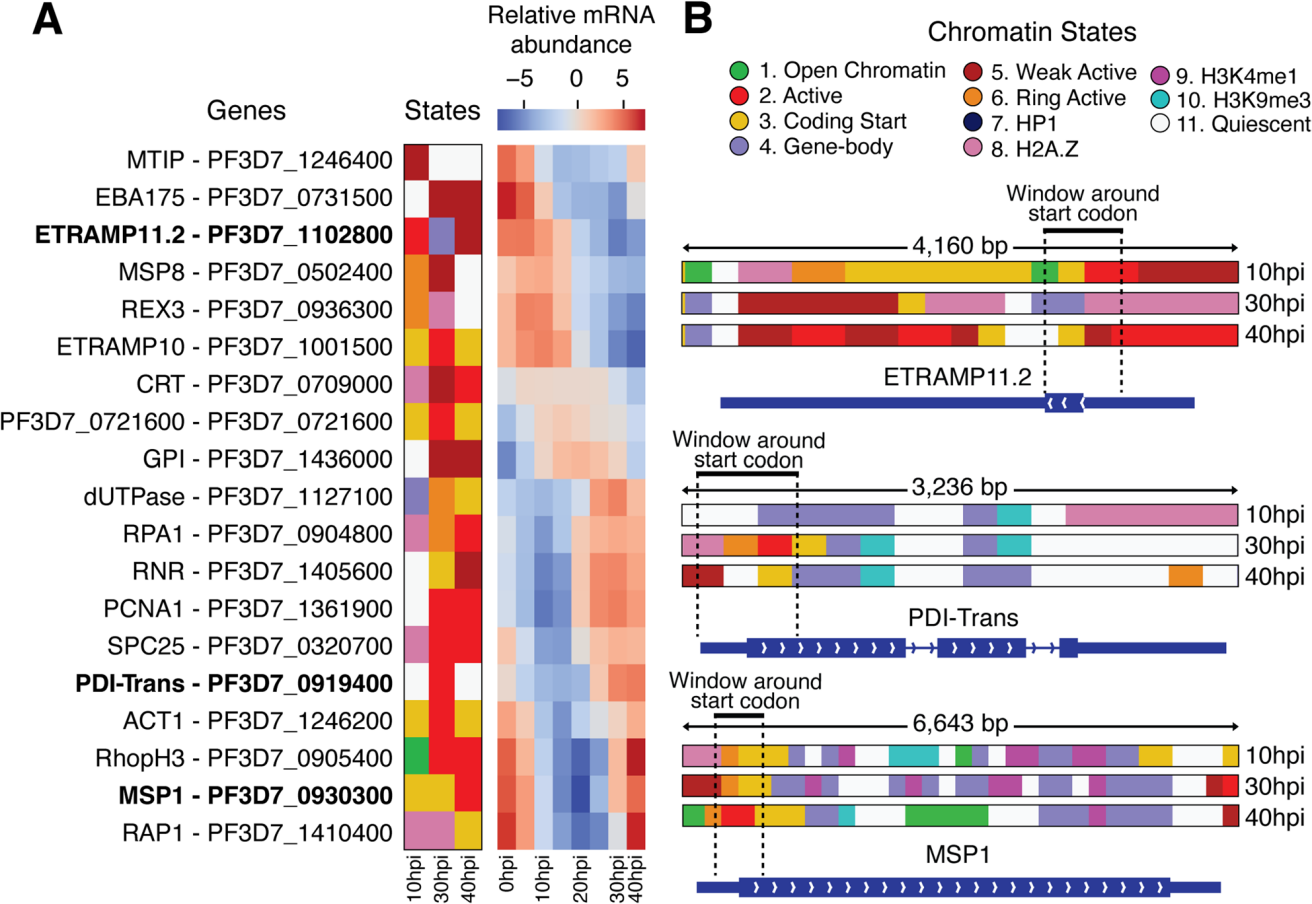
Associations between chromatin state transitions and transcript count dynamics for a selection of *P. falciparum* genes. **A** Representative chromatin states at start codons at three timepoints (left) and mRNA abundance heatmap from timepoints throughout the IDC (right; RNA-seq data sourced from [34]) for each of 19 selected genes. **B** Chromatin states for the three timepoints at ETRAMP11.2, PDI-Trans, and MSP1 gene loci. Thick blue bars at each gene indicate exonic regions, thin blue bars indicate UTR regions and chevrons indicate the orientation of transcription

To illustrate associations between chromatin states and mRNA dynamics for individual genes, we focused on a subset of genes that were selected based on their mRNA abundance dynamics at different stages of the IDC [71] (Fig. 4A). From this set, we highlight three representative examples (Fig. 4B) that peak in transcript counts at different stages of the IDC. The mRNA transcript encoding the early-transcribed membrane protein 11.2 (ETRAMP11.2, PF3D7_1102800) is most highly abundant during the ring stage, though its expression begins in late schizonts [34, 72]. The chromatin profile at its start codon shows the “Active” state 2 during the ring stage, transitioning to less active “Gene-body” state 4 in trophozoites, and then to “Weak Active” state 5 in schizonts, aligning well with its mRNA abundance pattern.

The gene encoding protein disulfide-isomerase PDI-Trans (PF3D7_0919400), which facilitates proper protein folding [73], has peak mRNA abundance in trophozoite and schizont stages. Its chromatin states transition from the “Quiescent” state 11 in ring stage to the “Active” state 2 in trophozoites and back to “Quiescent” state 11 in schizonts. Closer examination reveals some “Weak Active” state 5 and “Coding Start” state 3 signals near the start codon in schizonts, while the increasing presence of “Gene-body” state 4 from trophozoites to schizonts may indicate reduced transcription in the subsequent developmental stages.

Merozoite surface protein 1 (MSP1) (PF3D7_0930300) is one of the most highly abundant proteins on the merozoite surface [74–76]. MSP1-encoding mRNA transcripts increase beginning in the schizont stage while the parasite is still developing inside the host cell. Interestingly, the *msp1* gene displays the transcriptionally neutral “Coding Start” state 3 during ring and trophozoite stages before transitioning to the “Active” state 2 in schizonts, coinciding with its rapid increase in transcript levels. The RNA-seq data shows transcript counts beginning to increase in trophozoites, corresponding with the appearance of the “Weak Active” state 5 near the start codon. These examples show a positive correlation between mRNA transcript abundance dynamics for genes expressed throughout the IDC and changes in chromatin states.

Together, our analysis of temporal mRNA dynamics at both the global and individual-gene level demonstrate that chromatin state transitions are tightly coupled with the major shifts in transcript abundance required for parasite development.

### ApiAP2 transcription factors display distinct associations with chromatin states

**Fig. 5:**
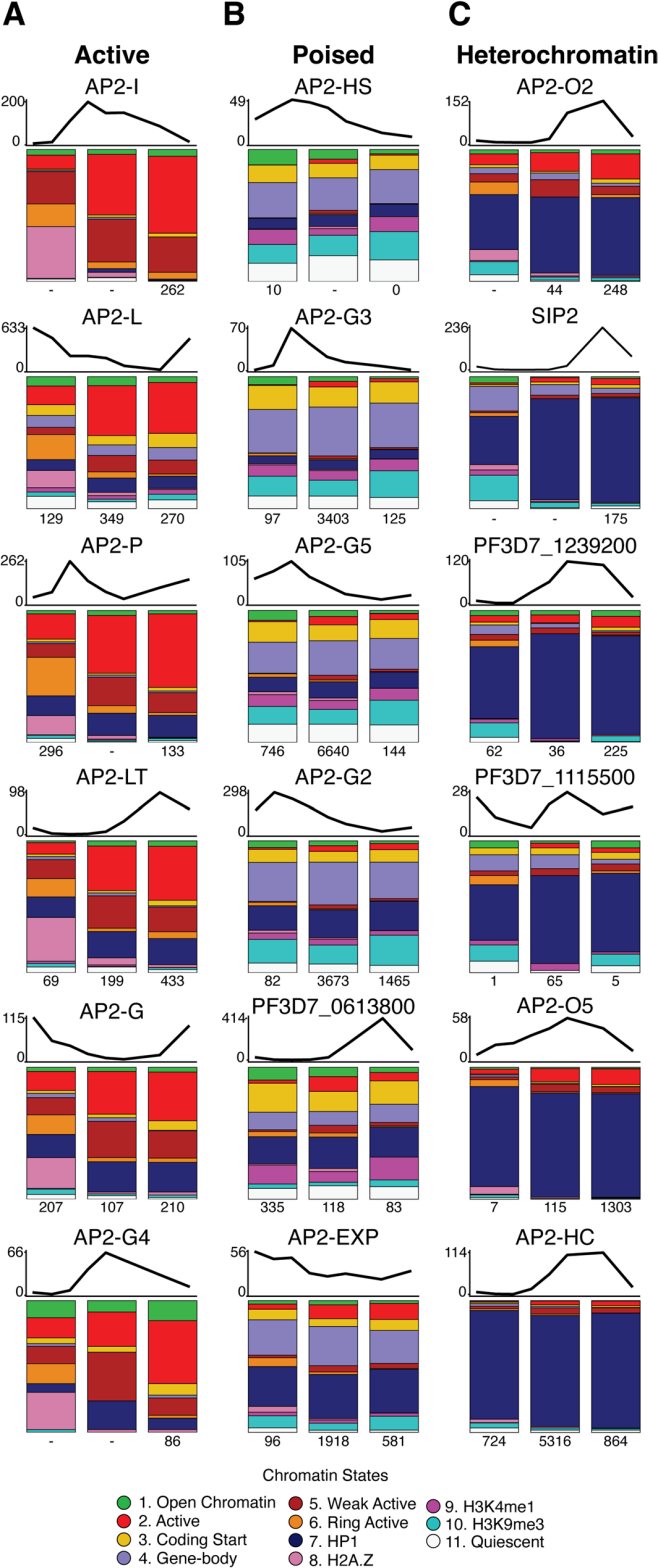
Bar plots show the relative frequencies of chromatin states at transcription factor binding sites across three developmental timepoints (10hpi, 30hpi, and 40hpi). For each transcription factor, all ChIP-seq peak locations were analyzed to determine the chromatin state at the peak summit. Above each set of bar plots, line graphs indicate the temporal mRNA abundance pattern of each transcription factor (FPKM values). The mRNA abundance of each factor is shown over eight timepoints from 5hpi to 40hhpi. Numbers below each bar represent the total count of ChIP-seq peaks identified for each transcription factor at the corresponding timepoint. **A** Transcription factors in the “active” category, which predominantly bind to promoter-associated chromatin states. **B** Transcription factors in the “poised” category, which associate with a mixture of chromatin states including potentially responsive regulatory regions. **C** Transcription factors in the “heterochromatin” category, which primarily bind to regions marked by the “HP1” state 7

Transcription factors can both influence and be influenced by chromatin states [77, 78]. In animal genomes, TFs display varied binding preferences depending on both DNA sequence and chromatin environment [79, 80]. While many TFs prefer to bind to active enhancers or promoters [81, 82], some are known to bind primarily in heterochromatic regions [83], and so-called pioneer TFs can actively promoter chromatin accessibility at their binding sites [84–86]. Relatively little is known about the chromatin state associations of TFs in *P. falciparum* [36]. To address this gap, we analyzed the genome-wide DNA-binding locations (based on ChIP-seq data) of 18 ApiAP2 transcription factors during the IDC. Our analysis revealed that these 18 TFs cluster into three distinct categories based on the patterns of chromatin states at their DNA binding sites (Fig. 5).

The first category (Fig. 5A) comprises six TFs (AP2-I, AP2-L, AP2-P, AP2-LT, AP2-G, and AP2-G4) that primarily bind to active, TSS-associated chromatin states, including “Active” state 2, “Weak Active” state 5, “Ring Active” state 6, and “H2A.Z” state 8. AP2-G and AP2-P exemplify this category of TFs; both are transcriptional activators associated with increased mRNA abundance of downstream genes [87–89]. Although AP2-P has been reported to exhibit some heterochromatin-adjacent binding [89], our analysis finds that the majority of AP2-P peaks lie in euchromatic promoter-associated states (specifically, “Active” state 2, “Weak Active” state 5, and “Ring Active” state 6). AP2-I, which is also considered a transcriptional activator, shows the strongest preference for “Active” state 2 and “Weak Active” state 5 within this group, consistent with its role in regulating genes related to cell division, growth, and invasion [90]. Furthermore, AP2-I is associated with the bromodomain protein BDP1, which binds acetylated histones at gene promoters consistent with the acetylation-rich “Active” state 2, “Weak Active” state 5, and “Ring Active” state 6 [90, 91].

The second category (Fig. 5B) includes six TFs (AP2-HS, AP2-G3, AP2-G5, AP2-G2, PF3D7_0613800, and AP2-EXP) associated with a more diverse set of chromatin states, including the “Coding Start” state 3, the potentially poised “Gene-body” state 4, “HP1” state 7, and “H3K9me3” state 10. Several TFs in this category, including AP2-G2 and AP2-G5, have been implicated in regulating the transition from asexual to sexual stage of development. Sexual development (or gametocytogenesis) also takes place in infected red blood cells and requires a 12-day maturation process to produce male and female gametes that will be infectious to mosquitoes during a bloodmeal. It has been proposed that AP2-G2 and AP2-G5 may act to repress the premature expression of key gametocyte genes [92, 93]. The most common chromatin states at binding sites in this category display varied relationships with mRNA abundance: “Coding Start” state 3 and “H3K9me3” state 10 do not strongly correlate with mRNA abundance, “Gene-body” state 4 associates with activation in trophozoites and schizonts, while “HP1” state 7 correlates with low mRNA abundance. Unlike the states preferred by the “active” TF category, the preferred chromatin states in this group maintain relatively stable abundance throughout the time course. Given the mixed association with mRNA abundance patterns, we characterize this set of TFs as “poised” in the IDC and potentially responsive to developmental or environmental signals.

Finally, we identified six TFs (AP2-O2, SIP2, PF3D7_1239200, PF3D7_1115500, AP2-O5, and AP2-HC) that are predominantly associated with the heterochromatin-associated “HP1” state 7 that is largely found in subtelomeric regions (Fig. 5C). Of particular interest in our heterochromatin associated factors is SIP2, which is known to bind at heterochromatin boundaries in telomeric regions [94]. A notable shift in chromatin states at SIP2 binding sites is seen following peak SIP2 mRNA abundance (Fig. 5C), transitioning from predominantly HP1-associated chromatin in trophozoite and schizont stages to a mixed set of states in ring stage (i.e., from 40hpi to 10hpi). This shift is remarkable given the overall stability of “HP1” state 7 across the genome, and may reflect SIP2’s crucial role in activating genes during merozoite formation following the schizont stage [95].

While all ApiAP2 family members contain one or more AP2 DNA-binding domains, little is known about how their non-DNA-binding domains contribute to the diversification of regulatory activities. The three categories of ApiAP2 TFs found here — “Active,” “Poised,” and “Heterochromatin” — may correspond to functional distinctions within the ApiAP2 protein family. Active TFs activate transcription and are linked to euchromatin; Poised TFs are likely repressors that bind intermediate chromatin states and gene bodies; and Heterochromatin TFs are involved in silencing genes, localizing to heterochromatin regions. Future studies are required to link these distinct chromatin associations to protein components.

## Discussion

In this study, we comprehensively characterized the chromatin landscape of asexual *P. falciparum* parasites by integrating a collection of diverse epigenomic datasets at several timepoints throughout the asexual intraerythrocytic developmental cycle. Using ChromHMM, we defined eleven distinct chromatin states based on combinatorial patterns of six functionally relevant histone modifications/variants, chromatin accessibility (ATAC-seq), and Heterochromatin Protein 1. Our analysis reveals that chromatin states are highly dynamic throughout the IDC, with approximately two thirds of the genome undergoing at least one chromatin state change between the three timepoints examined. These dynamic changes are closely associated with both lifecycle progression and mRNA abundance patterns, highlighting a complex interplay between chromatin structure and transcriptional regulation.

Several *P. falciparum* gene-body states are atypical of what has been reported in vertebrates, which associate H3K4me1 with enhancers [51, 52], and *Saccharomyces cerevisiae* (budding yeast) which lack both H3K9me3 and HP1 [96]. The potentially poised “Gene-body” state 4, combining H3K4me1 and H3K9me3 without HP1, is the dominant state at ~ 7–10% of genes and maintains a stable global abundance throughout the time course. It occasionally shifts to “Coding Start” state 3 (enriched in H3K4me1) or “H3K9me3” state 10, which are also present at exons. H3K4me1 enrichment at gene bodies has been reported in *Toxoplasma gondii* [97], and H3K4 methylation has also been reported over gene bodies in *S. cerevisiae* [98], suggesting that the presence of H3K4me1 at gene bodies may be more common in single celled eukaryotes. We propose that these combinations reflect unique poised or attenuated transcriptional states in exons that are distinct from H3K4me1’s canonical role at vertebrate enhancers.

The largest shift in chromatin states occurs during the transition from the ring stage to the trophozoite stages as the parasite shifts from initial invasion of the red blood cell to a highly metabolic state [19]. This transition involves a global gain of H3K4me3, shifting regions from “Ring Active” state 6 to “Active” state 2, and from “H2A.Z” state 8 to “Weak Active” state 5. These states (i.e., states 2, 5, 6, and 8), and the “H3K4me1” state 9 are highly dynamic, with frequent transitions to and from other states between timepoints. Meanwhile, the “HP1” state 7 and the “Quiescent” state 11 are highly stable. This suggest that there are regions of the malaria parasite genome that are stably regulated throughout asexual development versus those that are rapidly remodeled.

Temporal chromatin shifts are closely linked to the parasite’s dynamic pattern of mRNA abundance. While transitions to active, acetylated states (“Active” state 2, “Coding Start” state 3, and “Ring Active” state 6) at gene start codons correlate with increased transcription and shifts to H3K9me3-marked states (“Gene-body” state 4, “HP1” state 7, and “H3K9me3” state 10) correlate with mRNA abundance decline, other transitions revealed more complex relationships. For instance, gaining the “Weak Active” state 5 is paradoxically associated with reduced gene transcript counts, suggesting a nuanced role in potentially fine-tuning or dampening transcription at highly active loci.

The “HP1” state 7 is strongly correlated with maintained transcriptional repression throughout asexual development. This state is enriched at clonally variant genes in both sub-telomeric regions as well as chromatin-internal heterochromatic islands that generally include multiple copies of the *var* (encoding PfEMP1) and *rifin* gene families involved in immune evasion [99–102]. These genes typically display mutually exclusive expression patterns (i.e., one at a time) within individual parasites [62, 63], explaining their population-averaged association with the repressive “HP1” state 7.

By analyzing ApiAP2 transcription factor binding, we identified three distinct chromatin state associations. ApiAP2 TFs in the “Active” category, including AP2-I and AP2-P, predominantly bind to TSS-associated chromatin states (i.e., “Active” state 2, “Weak Active” state 5, “Ring Active” state 6, and “H2A.Z” state 8). These factors generally regulate invasion and pathogenesis genes [89, 90], with the notable exception being AP2-G, which controls gametocyte commitment [87, 88, 103, 104]. Their association with TSS-related chromatin states suggests they function primarily as transcriptional activators. While our temporal resolution cannot definitively determine whether these TFs require accessible chromatin or actively remodel chromatin themselves, the mRNA abundance dynamics of some TFs correlates with the timing of active state transitions at their binding sites.

The “Poised” TF category bind to more diverse chromatin states less strongly correlated with active transcription, including “Gene-body” state 4 and “H3K9me3” state 10. These TFs may function to attenuate gene expression without fully silencing genes through heterochromatin formation. Several TFs in this category, including AP2-G2 and AP2-G5, are thought to repress gametocyte-related genes [92, 93], potentially allowing for quicker transcriptional activation compared to fully heterochromatic genes.

The “Heterochromatin” category comprises TFs that primarily bind to sub-telomeric regions marked by “HP1” state 7, suggesting roles in maintaining heterochromatin or regulating clonally variant genes [32, 94]. Our categorization partially aligns with a previous study that defined eight ApiAP2 TFs as heterochromatin-associated factors based on correlation with HP1 binding [32]. Four of these (AP2-O2, PF3D7_1239200, AP2-O5, and AP2-HC) fall within our heterochromatin-binding category, while three others (AP2-G5, AP2-G2, and AP2-EXP) align with our “Poised” TF category. Interestingly, the remaining previously defined heterochromatin-associated factor, AP2-P, falls within the “Active” category in our analysis. However, consistent with the previous categorizations, we observe that subsets of binding sites for all of the listed factors overlap the “HP1” state 7.

A recent proximity-labeling proteomic mapping of chromatin environments revealed interactions spanning both heterochromatic and euchromatic contexts for several ApiAP2 TFs [105]. SIP2, AP2-HC, AP2-O2, AP2-LT, AP2-G5, and AP2-G2 were all associated with heterochromatin and euchromatin, confirming our observations. AP2-I was associated only with euchromatin, consistent with our “Active” category. AP2-EXP, AP2-L, AP2-O5, and AP2-P were only found to be associated with heterochromatin; while we categorize AP2-EXP, AP2-L, and AP2-P as generally non-heterochromatic, some binding sites for all three TFs overlap with “HP1” state 7.

Our findings are also complementary to a previous study in which a five-state ChromHMM model was used to investigate a limited set of histone marks (H3K27ac, H3K9ac, and H3K4me3) in egressing *P. falciparum* schizonts, blood cell invading merozoites, and early ring stage parasites [35]. Reers, et al., identified a promoter-associated state that has high signals for all three marks, aligning with “Active” state 2 in our study (Fig. 1). They also found that their promoter-associated state was highly prevalent in merozoites, a stage for which we did not include data. A second promoter-associated state from this study was characterized by high H3K9ac and H3K4me3 but low H3K27ac and is consistent with “Weak Active” state 5 in our model. This state was prevalent at promoters in schizonts, but less so in merozoites. Additionally, they identified a state with high H3K9ac signals that was enriched in ring stage parasites but not merozoites, which is likely consistent with our “H2A.Z” state 8. Overall, our study and that of Reers, et al., provide consistent views of chromatin regulatory dynamics at different developmental stages. Our study focuses on the IDC, while they focused on egress and reinvasion, demonstrating the value of a more comprehensive, integrated chromatin state analysis across all stages of the malaria parasite lifecycle.

Although our work offers new insights into the chromatin landscape of *P. falciparum*, several limitations should be noted. The epigenomic datasets originated from different laboratories [24, 26, 28, 32–34], introducing potential variability despite our efforts to standardize analysis pipelines. Our analysis is restricted to available histone modifications at three timepoints, potentially missing important modifications or finer temporal dynamics. Due to the inherent challenges associated with matching the precise staging of synchronized parasites and aligning data from multiple labs, some observed chromatin state patterns may reflect lifecycle stage differences between different labs or other batch effects. Future work would benefit from a standardized, comprehensive, epigenomic profiling at high temporal resolution across the complete lifecycle. In addition, perturbation studies of histone modifiers and/or the TFs themselves will more clearly define the causal relationships between ApiAP2 TFs and chromatin signals at their binding sites.

Our application of chromatin state analysis to the malaria parasite *Plasmodium falciparum* highlights the dynamic nature of the chromatin landscape during the IDC. These data also define distinct categories of ApiAP2 TFs based on their chromatin binding preferences. Together, our findings indicate that *P. falciparum* TFs not only recognize specific DNA sequences but also show differential preferences for chromatin landscapes, potentially explaining their target specificity through a combined recognition mechanism. Our findings provide a foundation for future studies aimed at understanding gene regulation in this important human pathogen.

## Methods

### Data sources

Nine data types were used to construct chromatin states in this study: ChIP-seq for H3K9ac [24], H3K4me1 [33], H3K4me3 [24], H2A.Z [33], H3K27ac [33], H3K18ac [33], H3K9me3 [28], HP1 [26, 32], and ATAC-seq [34]. The HP1 datasets were processed and aligned separately and were then merged internally by ChromHMM. The mRNA abundance data was derived from RNA-seq datasets published by Toenhake, et al., [34], which was collected in parallel with the ATAC-seq data used in this study.

Most datasets used for chromatin state definition included samples from all three timepoints (10hpi, 30hpi, and 40hpi), with the exception of H3K9me3, for which data were only available at 20hpi and 40hpi. To maintain consistency across all features, the 20hpi H3K9me3 data were used to represent both the 10hpi and 30hpi timepoints in the model. This approach was supported by the high correlation between the available H3K9me3 timepoints (Pearson correlation *r* = 0.992 using 10 kb bins; Supplemental Figure S4), suggesting minimal temporal variation in this mark. We excluded datasets with non-overlapping timepoints and any datasets that did not use the 3D7 *P. falciparum* strain. We also prioritized datasets that profiled multiple marks to minimize cross-study variance.

The majority of ApiAP2 TF ChIP-seq datasets (AP2-L, AP2-G4, AP2-HS, AP2-G3, AP2-G5, PF3D7_0613800, AP2-O2, PF3D7_1239200, PF3D7_1115500, and AP2-O5) were obtained from Shang, et al., [32]. Several TFs were profiled by multiple laboratories, and we used merged peak sets in these cases: AP2-G2 [32, 93], AP2-P [32, 89], AP2-LT [32, 36], AP2-EXP [32, 106], and AP2-HC [26, 32]. Three additional ChIP-seq datasets were also used: AP2-I [90], AP2-G [87], and SIP2 (GEO ID: GSE296868).

Data sources are summarized in Supplemental Table 1.

### Data acquisition and preprocessing

A standardized reprocessing pipeline was used for all datasets to ensure consistency. Raw sequencing reads (fastq files) were quality-trimmed using Trimmomatic (v0.39) with parameters SLIDINGWINDOW:4:30 MINLEN:35 [107]. Trimmed reads were aligned to the *P. falciparum* 3D7 reference genome using BWA-MEM (v0.7.17) with the -M option and filtered for alignment quality (minimum Phred score of 30) using Samtools (v1.16.1) [108, 109]. Duplicate reads were removed using Picard’s MarkDuplicates tool (v2.24.1) with parameters REMOVE_DUPLICATES = true and VALIDATION_STRINGENCY = STRICT [110]. After duplicate removal, biological replicates were merged. For ChIP-seq data, peak calling was performed using MACS2 (v2.2.7.1) with parameters -g 2e + 7 -q 0.001 --nomodel --shift 0 --extsize 200 [111].

RNA-seq reads were trimmed with Trimmomatic (v0.39) using parameters SLIDINGWINDOW:4:30 MINLEN:35, then aligned using STAR (v2.7.10b) with parameters --runMode alignReads --genomeLoad NoSharedMemory --readFilesCommand zcat --outSAMtype BAM Unsorted --alignIntronMax 10,000 --alignIntronMin 30 [112]. Gene-level transcript counts were generated using HTSeq-count (v2.0.3) with parameters --mode union --format bam --order name --idattr gene_id --minaqual 20 --stranded = no --type exon [113], and converted to Fragments Per Kilobase of exon per Million mapped reads (FPKM) values.

To ensure consistency in timepoint labeling across datasets, we adjusted timepoint labels where possible by aligning RNA-seq data across studies and by comparing stage-specific gene mRNA abundance profiles with asexual blood stage labels from PlasmoDB.org for several periodically expressed genes [71]. Specifically, the datasets from Toenhake, et al., [34] appeared shifted forward compared to the reference datasets on PlasmoDB as exemplified by pairwise Pearson correlation on normalized counts (FPKM values) with the RNA-seq dataset by Chappell, et al., 2020 [21]. To correct this discrepancy, we adjusted the labels in the Toenhake, et al., ATAC-seq and RNA-seq timepoints by 5hpi so that 15hpi was renamed to 10hpi, 35hpi to 30hpi, etc. The 40hpi schizont timepoint was not adjusted as it aligned with the latest timepoint in the reference dataset and maintained a consistent schizont stage measurement.

### Chromatin state modeling

ChromHMM (version 1.23) was used to call states, with each developmental timepoint treated as a distinct cell type in the ChromHMM framework [2]. For each chromatin feature, BAM files were binarized using ChromHMM’s BinarizeBam function with a bin size of 200 bp and using appropriate control files for each experiment. This function creates a table of presence/absence for each mark in each bin. The LearnModel function was then applied to construct the chromatin state models.

To determine the optimal number of chromatin states, models with 4 to 25 states were generated and evaluated based on several criteria: the percentage of genome covered by the quiescent state; the number of states with similar emission profiles; and the genomic coverage of the least frequent state. As the number of states was increased, the proportion of genome classified as quiescent decreased non-linearly. The “elbow point” in this relationship was identified, beyond which additional states primarily subdivided existing categories rather than identified truly distinct chromatin types (Supplemental Figure S5). By analyzing correlations between emission profiles across models with different numbers of states, it was determined that 11 states optimally captured chromatin diversity while minimizing redundancy.

We also assessed the effect of varying the bin size on chromatin state analyses by running ChromHMM with bin sizes of 500 bp or 1,000 bp (Supplemental Figure S6). Many of the chromatin states discovered using ChromHMM have similar emission probabilities across 200 bp, 500 bp, and 1,000 bp bin sizes (Supplemental Figure S6A-C), suggesting that these chromatin state patterns are robust to the bin size parameter. The temporal state transition probabilities for equivalent chromatin states are also similar (Supplemental Figure S6D-F). However, as the bin size is increased, the chromatin state emission probabilities lose definition and some chromatin states discovered at 200 bp resolution are lost from the analysis. For example, the 500 bp bin size chromatin states do not contain a state equivalent to the “H2A.Z” state 8 but instead contain a state with a mixture of signals for H3K4me3, H3K9ac, and H2A.Z. Such mixed signal states are also prevalent in the 1,000 bp bin size ChromHMM results, suggesting that they are due to artifactual smoothing across neighboring chromatin signals as the bin size is increased. We therefore used a bin size of 200 bp for our chromatin state analyses in this study. This bin size is also the default parameter in ChromHMM and is consistent with the 200 bp bin size used by almost all major applications of ChromHMM [4, 7, 10, 13, 35, 59, 61, 114–121], including the application of ChromHMM in the Human ENCODE project [13], RoadMap Epigenomics [10], Mouse ENCODE [61], Drosophila modENCODE [4], and FAANG [117, 120].

The relative feature enrichments shown in Fig. 1B were calculated using the formula implemented in ChromHMM’s OverlapEnrichment function. This formula computed the ratio of observed overlap to expected overlap (assuming independence between states and genomic features), providing a measure of association strength. The enrichment values were normalized column-wise to highlight which chromatin state showed the strongest association with each genomic feature.

### Associations between mRNA abundance and chromatin states

To assess gene mRNA dynamics and chromatin state dynamics (Fig. 3), states were assigned to genes based on the predominant chromatin state near the start codon of the gene. For each gene, the most frequent chromatin state in a 600 bp window around the start codon was identified. If more than one state tied for the most overlap, measured in bases, one of the highest tied states was randomly selected.

To associate chromatin states with mRNA abundance (Fig. 1E), each gene was assigned to a state if the gene contained the state or if the state was within 2 kb upstream of the gene’s transcription start site (TSS). In cases where two genes were nearby, the closest TSS was used. For overlapping genes, both genes were assigned to the state. The average gene transcript count associated with each state was calculated and presented in Fig. 1E to represent state-centric mRNA abundance associations.

### Associations between transcription factor binding sites and chromatin states

For each TF, the union of ChIP-seq peaks across timepoints was taken, and bedtools intersect (v 2.30.0) was used to filter out overlapping peaks to avoid overcounting [122]. Pybedtools (v 0.9.0) [123] was used to compare TF binding sites to chromatin state locations across all three timepoints. Importantly, chromatin states can still be examined at transcription factor binding sites even when ChIP-seq data is not available for a particular time point, as peak coordinates allow for the analysis of chromatin states at those coordinates across all three time points. All results are displayed as proportions in Fig. 5, with each column representing the proportion of the union of TF binding sites covered by each chromatin state at each of the three timepoints. All TF chromatin state associations were determined to be statistically significant by a chi-squared test.

## Supplementary Information

Supplementary Material 1.

## Data Availability

BED files containing chromatin state tracks for each timepoint are available at https://github.com/seqcode/pfal-chrom-states-trackhub. A browsable set of chromatin state and associated data tracks are available via the UCSC genome browser: http://genome.ucsc.edu/cgi-bin/hgTracks?db=GCF_000002765.5&hubUrl=https://seqcode.github.io/pfal-chrom-states-trackhub/trackhub/hub.txt.
