# Supplementary material for: Chromatin state dynamics during the Plasmodium falciparum intraerythrocytic development cycle
Source: BMC Genomics. 2026 Jan 7;27:142. doi: 10.1186/s12864-025-12455-3 (PMC12870380; doi:10.1186/s12864-025-12455-3)
Supplement: Supplementary file 1 — Supplementary Material 1. [file 12864_2025_12455_MOESM1_ESM.pdf]

SUPPLEMENTARY DATA FOR:

Chromatin state dynamics during the *Plasmodium falciparum* intraerythrocytic development cycle

Alan S. Brown Jr.[1,2], Manuel Llinás[1,2,3*], Shaun Mahony[1*]

1 Center for Eukaryotic Gene Regulation, Department of Biochemistry & Molecular Biology, The Pennsylvania State University, PA 16802, USA
2 Huck Center for Malaria Research, The Pennsylvania State University, PA 16802, USA
3 Department of Chemistry, The Pennsylvania State University, PA 16802, USA

* To whom correspondence should be addressed: mahony@psu.edu; manuel@psu.edu

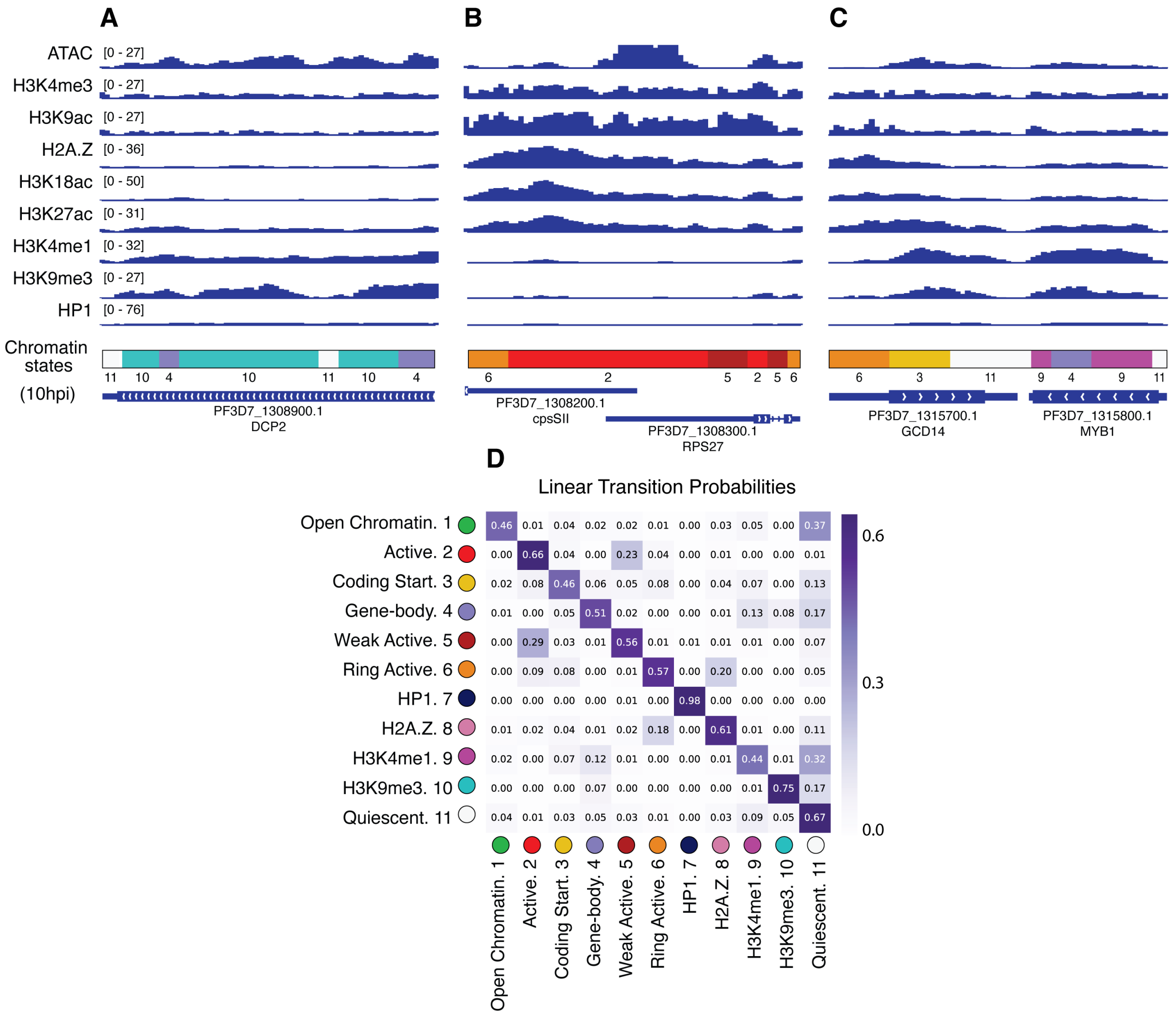

Supplemental Figure S1. A) 10hpi chromatin signal tracks and associated chromatin states in a region showcasing state 10 at coordinates Pf3D7_13_v3:408,432-411,760. **B)** 10hpi chromatin signal tracks and associated chromatin states in a region showcasing states 2 and 5 at coordinates Pf3D7_13_v3:369,395-372,723. **C)** 10hpi chromatin signal tracks and associated chromatin states in a region showcasing state 9 at coordinates Pf3D7_13_v3:658,006-661,334. **D)** Linear transition probabilities (i.e., state transition probabilities between neighboring genomic bins) for the 11 chromatin states used in this paper.

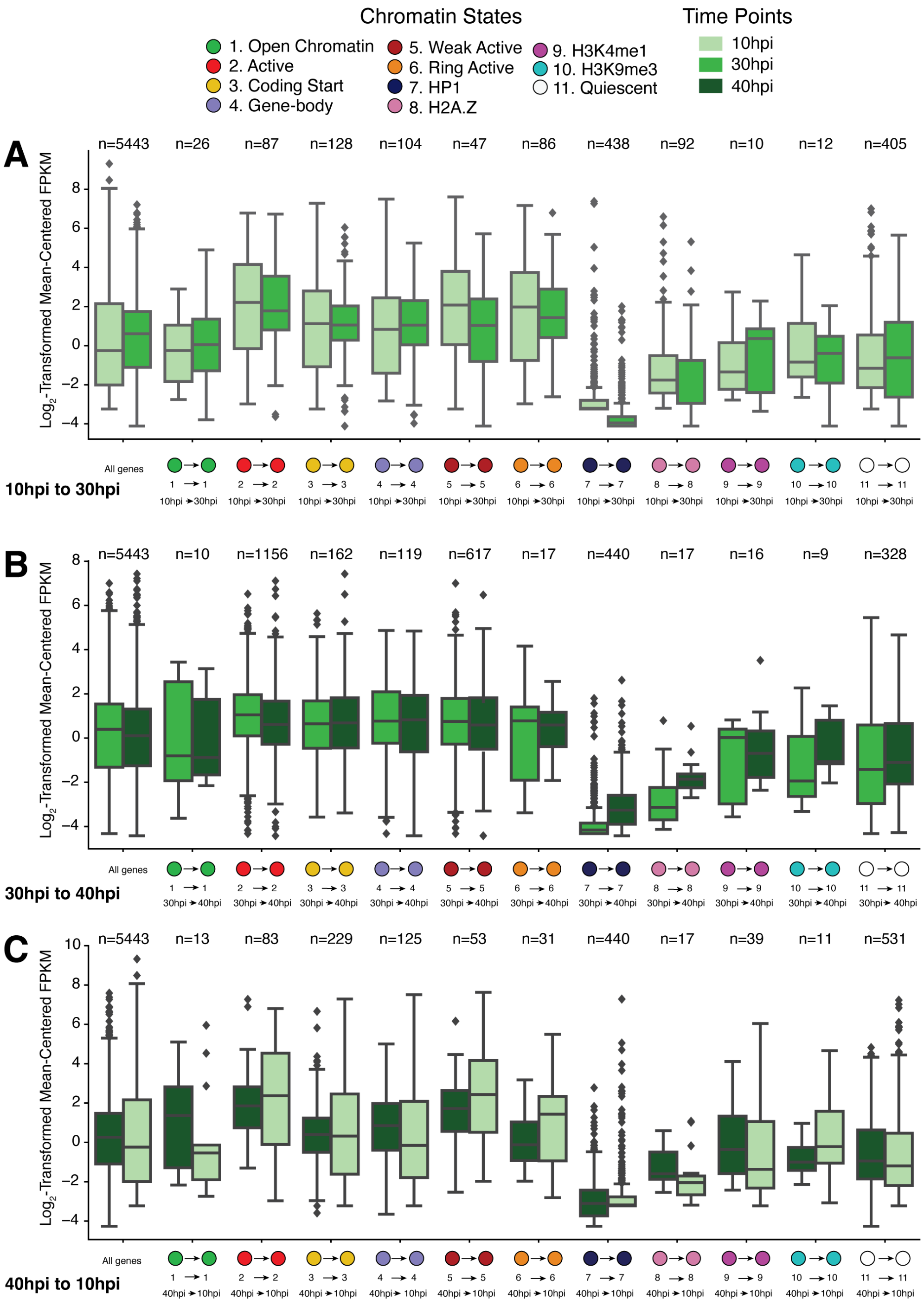

Supplemental Figure S2. Associations between chromatin state transitions and mRNA abundance dynamics at genes that do not change states between consecutive pairs of timepoints. Boxplots display the distributions of log_2-transformed mean-centered FPKM normalized RNA-seq values for groups of genes that display indicated self-transitions for each labeled state at the labeled timepoints. **A)** Chromatin state self-transitions and FPKM values from 10hpi to 30hpi. **B)** Chromatin state self-transitions and FPKM values from 30hpi to 40hpi. **C)** Chromatin state self-transitions and FPKM values from 40hpi back to 10hpi.

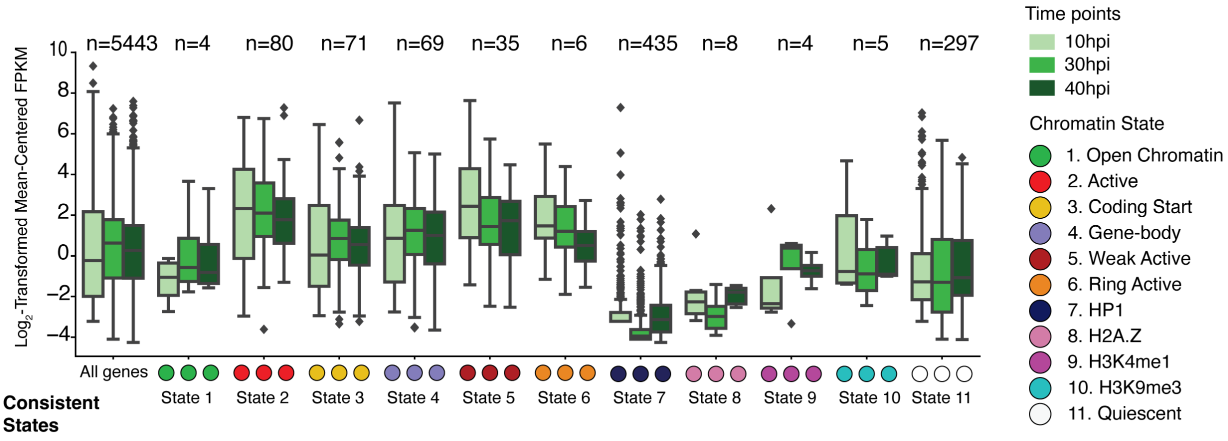

Supplemental Figure S3. Associations between chromatin state transitions and mRNA abundance dynamics at genes that never change states across all three timepoints. Boxplots display the distributions of log_2-transformed mean-centered FPKM normalized RNA-seq values for groups of genes that display indicated maintained states for each labeled state at the labeled timepoints.

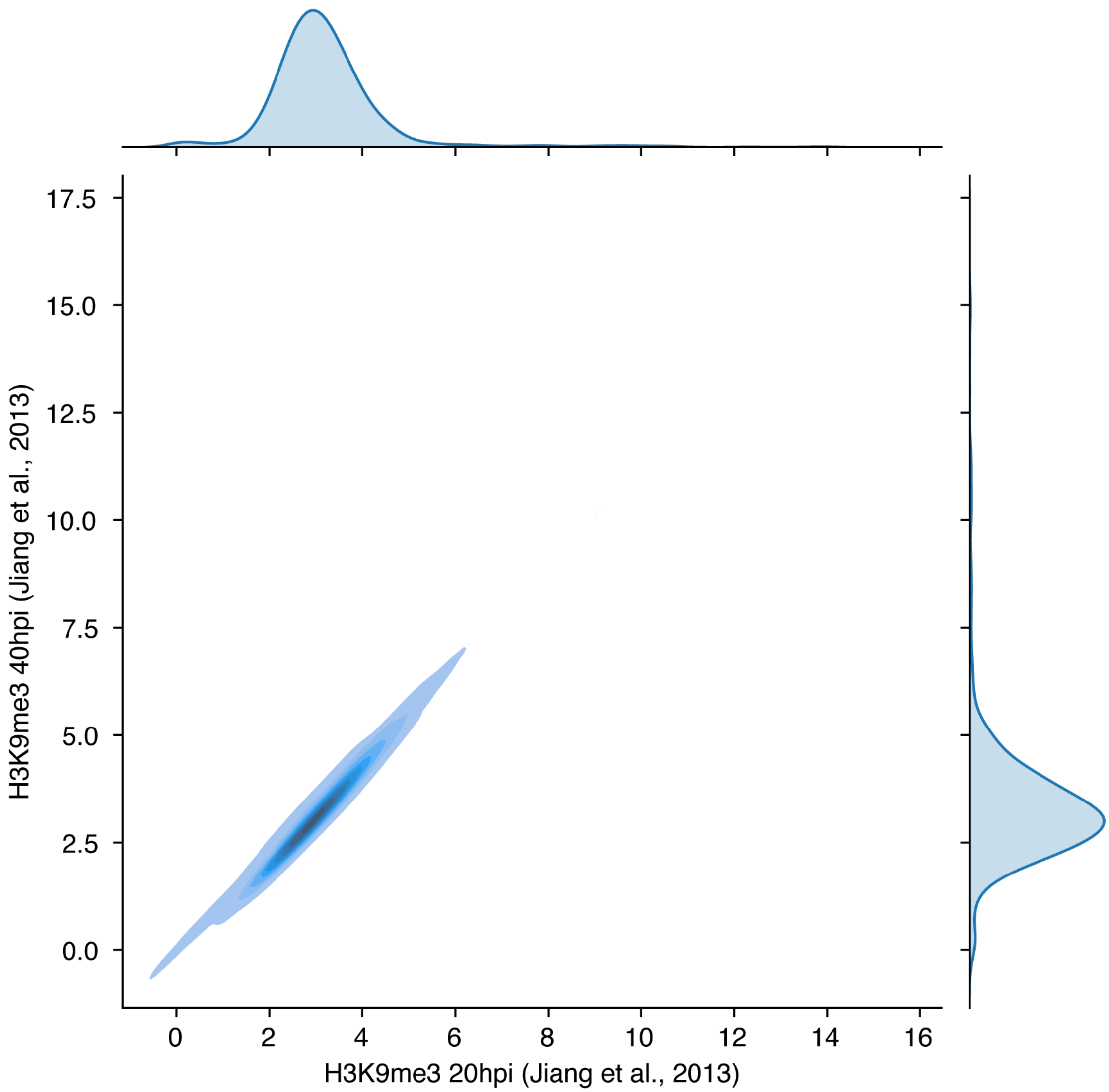

Supplemental Figure S4. Density plot of binned ChIP enrichment levels between the two H3K9me3 datasets from [28], justifying use of the 20hpi timepoint for both 10hpi and 30hpi timepoints in the chromatin state analysis. Pearson correlation coefficient = 0.992. The density plot and correlation are based on 10kbp bins across the genome using raw read counts.

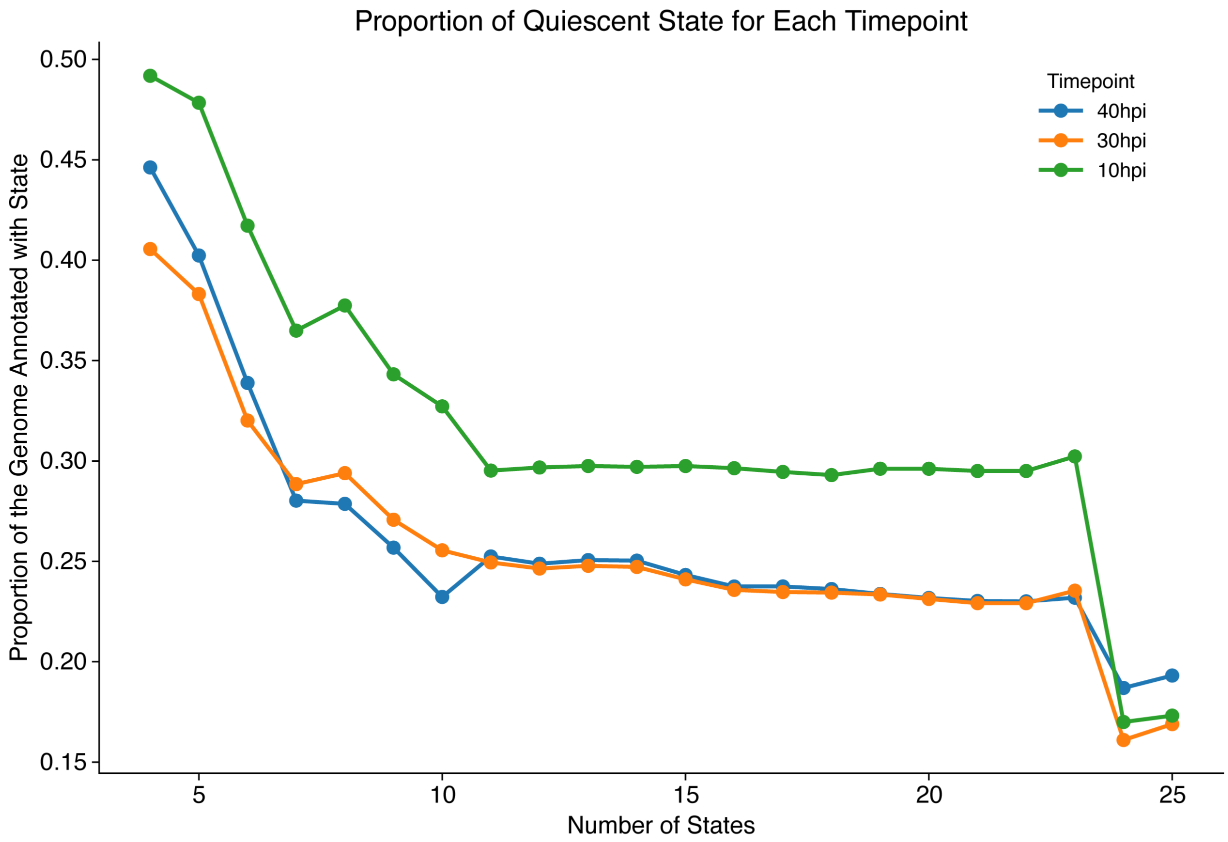

Supplemental Figure S5. Genome wide proportion of the quiescent state for ChromHMM models varying by the number of chromatin states generated from 4 states to 25 states. All three timepoints are shown for each number of states. The “elbow point” occurs at state 11.

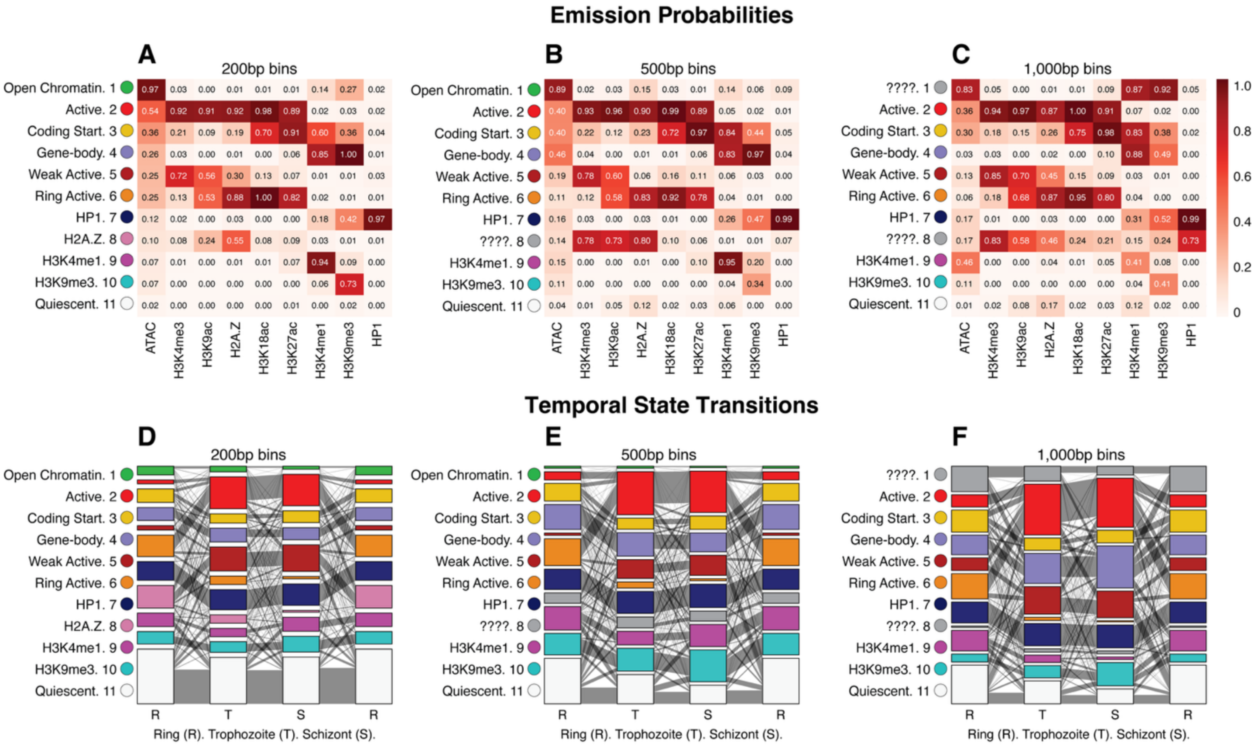

Supplemental Figure S6. Assessing the effect of varying the bin size on ChromHMM analyses. Hidden Markov Model emission probabilities (i.e., the probability of seeing the mark given the chromatin state) for each of the 11 chromatin states discovered by ChromHMM using 200bp (**A**), 500bp (**B**), and 1,000bp (**C**) bin sizes. Temporal chromatin state transitions across all genomic bins using 200bp (**D**), 500bp (**E**), and 1,000bp (**F**) bin sizes. Stacked bar graphs show the relative proportions of the 11 chromatin states during 10hpi ring (R), 30hpi trophozoite (T), and 40hpi schizont (S) stages.

Supplemental Table 1: Datasets, references, and accession numbers used in this study.

Data	Reference	GEO ID
ATAC-seq and RNA-seq	[34]	GSE104075
H3K9ac	[24]	GSE23787
H3K4me1	[33]	SRP252482
H3K4me3	[24]	GSE23787
H2A.Z	[33]	SRP252482
H3K27ac	[33]	SRP252482
H3K18ac	[33]	SRP252482
HP1	[26,32]	GSE154840, GSE184659
H3K9me3	[28]	SRP022761
AP2-I	[90]	GSE80293
AP2-L	[32]	GSE184659
AP2-P	[32,89]	GSE190497
AP2-LT	[32,36]	GSE184659, GSE212052
AP2-G	[87,92]	GSE120448, GSE149774
AP2-G4	[32]	GSE184659
AP2-HS	[32]	GSE184659
AP2-G3	[32]	GSE184659
AP2-G5	[32,92]	GSE184659, GSE149774
AP2-G2	[32,93]	GSE184659, GSE157753
PF3D7_0613800	[32]	GSE184659
AP2-EXP	[32,106]	GSE184659, PRJNA818769
AP2-O2	[32]	GSE184659
SIP2	Till Voss and Richard Bartfai	GSE296868
PF3D7_1239200	[32]	GSE184659
PF3D7_1115500	[32]	GSE184659
AP2-O5	[32]	GSE184659
AP2-HC	[26,32]	GSE184659, GSE154840
